# Extrapyramidal side effects and suicidal ideation under fluoxetine treatment: a case report

**DOI:** 10.1186/1744-859X-9-5

**Published:** 2010-01-18

**Authors:** Christos Christodoulou, Athanasia Papadopoulou, Emmanouil Rizos, Kalliopi Tournikioti, Xenia Gonda, Athanassios Douzenis, Lefteris Lykouras

**Affiliations:** 1 Second Department of Psychiatry, Athens University Medical School, 'Attikon' General Hospital, Athens, Greece; 2 Department of Clinical and Theoretical Mental Health, Kutvolgyi Clinical Center, Semmelweis University, Faculty of Medicine, Budapest, Hungary

## Abstract

**Background:**

We present the case of a 52-year-old woman with depression who developed extrapyramidal symptoms (mainly parkinsonism) and suicidal ideation while on fluoxetine.

**Methods:**

The patient underwent neurological and neuroimaging examination.

**Results:**

The patient's neurological and neuroimaging examinations were normal and there was no other cause of extrapyramidal symptoms. The patient showed remission of the aforementioned symptomatology when fluoxetine was discontinued.

**Conclusions:**

This case shows that fluoxetine can be associated with extrapyramidal symptoms, and this may have an aggravating affect on clinical depression progress and the emergence of suicidal ideation.

## Background

Extrapyramidal symptoms (EPSs) are an uncommon side effect of serotonin reuptake inhibitors (SSRIs). Concomitant use with antipsychotic medication or the presence of other risk factors (age, gender) increase the vulnerability to EPSs [1]. The most common EPS associated with SSRIs seems to be akathisia, followed by dystonia and parkinsonism. Fluoxetine is the SSRI most associated with extrapyramidal reactions in the majority of cases [2,3]. Even, in adolescents treated with fluoxetine, EPSs have been reported [4]. The symptoms are reversible with dose reduction, drug discontinuation, or by the addition of another agent, such as anticholinergic agents, β-blockers or benzodiazepines [5,6]. EPSs, and especially akathisia, have been associated with the emergence of suicidal ideation and suicidal acts in patients receiving fluoxetine [7,8], however, a clear relationship between fluoxetine and emerging suicidality is not certain [9].

We present the case of a female receiving fluoxetine who, after approximately 1 month of therapy, developed severe EPSs, mainly parkinsonism, with simultaneous emergence of suicidal ideation.

## Case presentation

A 52-year-old married woman presented to the emergency department of the Psychiatric Clinic of 'Attikon' General Hospital in Athens with depressive symptoms. The symptoms had begun 1 month prior to the referral to our hospital, and included: depressive mood, insomnia, early morning waking, loss of appetite and psychomotor retardation. At that time she visited a private psychiatrist, and was given antidepressant therapy with fluoxetine, 60 mg per day and alprazolam 1.5 mg daily for 4 weeks with no significant improvement. During her examination, she was obviously very depressed and anxious with no psychotic symptoms and she reported suicidal ideation for the last 10 days.

Her neurological examination revealed severe rigidity and bradykinesia. Her face was like a 'mask' and her facial movements were almost non-existent. She was speaking at low volume but she had no rest or movement tremor, and no severe instability. Clinically, although her treatment did not include any antipsychotic drugs, her appearance did not differ from typical antipsychotic-induced parkinsonism. Her husband had observed reduced facial expression for the last 2 weeks. The patient herself associated her suicidal ideation with the subjective sensation of a progressive movement disability. Her medical history revealed no neurological diseases. A magnetic resonance imaging (MRI) scan showed no abnormal findings.

It was recommended that fluoxetine treatment be stopped and alprazolam continued.

## Results

At 1 week later the improvement of her rigidity and bradykinesia was impressive. Her facial expression was almost normal. The emotional distress was reduced and surprisingly her suicidal ideation diminished with fluoxetine withdrawal. At that time she was given another class of antidepressant, showing significant improvement of her depression.

## Discussion

Considerable controversy exists regarding the relationship between fluoxetine and the emergence of suicidal ideation. Our patient had no neurological history. EPSs, mainly parkinsonism, had appeared in the last 2 weeks while she had been under medication with fluoxetine. Several reports published in the 1990s suggested that fluoxetine could be responsible for suicidal ideation or behaviour [7,10-12]. More recent studies have suggested this relationship as well. In a case-control analysis an association has been found between SSRIs and suicide events [13]. Moreover, a systematic review of randomised controlled trials supports the relationship between the use of SSRIs and the increased risk of suicidal behaviour. According to this review, such risk may be underestimated due to a number of methodological limitations of the published reports [14].

By contrast, the results of a recent cohort study based on a large sample do not support the hypothesis that treatment with SSRIs increases the risk of suicide [15]. In another matched case-control study, the risk of suicidal behaviour was reported to be similar among users of amitriptyline, fluoxetine and paroxetine [16]. Also, a nationwide cohort study in Finland found that fluoxetine was associated with a lower risk of suicide among different classes of antidepressants. Moreover, the results of the same study indicate that the current use of any antidepressant among suicidal patients was associated with increased risk of attempted suicide by self-poisoning, but with a decreased risk of completed suicide and death. The latter could be attributed to the fewer cardiovascular and cerebrovascular side effects of SSRI medication [17]. Finally, a meta-analysis of randomised control trials of SSRIs conducted by pharmaceutical companies indicated that there is evidence of increased risk of non-fatal self-harm in adults treated with SSRIs but no evidence of increased risk of suicidal thoughts [18].

Akathisia or dysphoric extrapyramidal reactions may be a contributing factor to the emergence of suicidal ideation during treatment with fluoxetine [19]. In our case, parkinsonism and restlessness (but no akathisia) were prominent in the clinical picture of the patient.

EPSs may have accidentally appeared in our patient, or an underlying organic disorder may have predisposed her to EPSs. However, an MRI scan revealed no abnormal findings that could explain a possible vulnerability to side effects. After stopping fluoxetine, the patient's extrapyramidal symptomatology improved dramatically as did her suicidal thoughts. This report suggests that severe EPSs, and in particular parkinsonism due to the implicated movement disability, cause distress, which in turn may cause suicidal ideation.

## Conclusions

It is possible that SSRI as monotherapy could be the cause of EPSs and subsequent suicidal ideation. Psychiatrists must be aware of this side effect and be prepared for clinical features characterised by movement disorders in patients treated with SSRIs.

## Consent

Written informed consent was obtained from the patient for publication of this case report and any accompanying images. A copy of the written consent is available for review from the Editor-in-Chief of this journal.

## Competing interests

The authors declare that they have no competing interests.

## Authors' contributions

CC made substantial contributions to the conception and design of the present study, and was also involved in drafting and revising the manuscript and gave final approval for the manuscript to be published. AP was codesigner of the present study and made substantial contributions in the acquisition, analysis and interpretation of the data. ER made substantial contributions in drafting the manuscript and revising it critically for intellectual content. KT contributed to the clinical evaluations and manuscript drafting. XG and AD made substantial contributions in revising the manuscript. LL made substantial contributions in drafting the manuscript and revising it critically for intellectual content, and gave final approval for the manuscript to be published. All authors read and approved the final manuscript.
